# The central role of laboratory medicine as the integrating element in healthcare services

**DOI:** 10.1186/1878-5085-5-S1-A131

**Published:** 2014-02-11

**Authors:** Olga Golubnitschaja, Ian D Watson, Vincenzo Costigliola

**Affiliations:** 1 European Association of Predictive, Preventive and Personalised Medicine, Brussels, Belgium; 2 European Federation of Clinical Chemistry and Laboratory Medicine, Milano, Italy

## 

The authors consider acute problems in the quality and management of medical services challenging health care systems worldwide. This actuality has motivated the representatives of the European Association of Predictive, Preventive and Personalised Medicine and European Federation of Clinical Chemistry and Laboratory Medicine to consider the efforts in promoting an integrative approach based on multidisciplinary expertise to advance health care. The position paper of EPMA and EFLM [1] provides a global overview of the problems related to medical services: pandemic scenario in the progression of common chronic diseases, delayed interventional approaches of reactive medicine, poor economy of health care systems, lack of specialised educational programmes, problematic ethical aspects of treatments as well as inadequate communication among professional groups and policy makers. The common position is focused on the patients' needs, expert recommendations for the relevant medical fields and plausible solutions which have a potential to advance health care services if the long-term strategies were to be effectively implemented as proposed in the position paper.

In particular, the paper makes following statements:

➣ Since chronic pathologies are generally triggered at the molecular level with consequent symptomatic manifestation of the disease, a laboratory based detection of pathology-specific molecular patterns would create a well-founded basis for the desirable predictive medical services giving the opportunity for optimal health care. This requires the application of innovative biotechnologies to predict human pathologies, the devising of appropriate and timely preventive strategies and individualised treatment planning.

➣ Multimodal diagnostics represents a model-based examination procedure with several levels of examination resulting in extended patient profiles and medical records which obligate inclusion of an interview with the patient/a questionnaire filled in for relevant information on any known pathology, medical imaging, laboratory diagnostics and evaluation of relevant risk factors. For laboratory diagnostics, it is highly recommended to use minimally invasive validated blood tests for the detection of stage-specific molecular patterns at complementary levels of targeted regulation (DNA polymorphisms, transcripts, protein expression, posttranslational modification, stage-specific sub-cellular imaging, shifted enzymatic activity etc.).

➣ Shifting the role of laboratory from the ‘passive performing’ to the ‘active advising’ is the next paradigm change in health care. This reconsideration of the laboratory-clinician interface might significantly advance the quality of current medical services, although the implementation of this approach across countries should be adapted to local conditions.

➣ The globalisation of markets and laboratory related business requires the comparability, or the unification, of laboratory test values. The mobility of both patients and health care professionals as well as the increasing global data flow require such comparability. Hence, the unification of laboratory tests should be placed at the top of the list of corresponding adapting measures.

➣ A new generation of ‘point-of-care’ monitoring devices is required. These mobile health technologies must enable both the remote management of the analytical process and the active engagement of laboratory professionals at the clinical level.

➣ Regarding the promotion of the concepts of ‘participatory’ medicine, people need to be advised of reliable information sources that are well adapted to a corresponding level of understanding (categories of children, youth and adults) and concrete interests of subpopulations (level of education, groups of professionals, patient cohorts). In the field of education, laboratory medicine may play a leading role providing up-to-date information that is accessible to the layman on laboratory tests and their interpretation for individual health and disease conditions. A professional version will enable for a detailed knowledge about bioactive molecules, enzymatic reactions, molecular and cellular processes which underlie the pathomechanisms of individual predispositions and pathologies as well as medical treatments.

➣ A creation of innovative medical records should be considered as a priority for scientific programmes of multidisciplinary character. An integrative bioinformatics is considered as the powerful tool to fulfil this highly ambitious task.

➣ Progressing from ‘disease care’ to ‘health care’ means a mandated implementation of a new philosophy of the well-being concept tailored to the person (age, gender, socio-economic status, individual predispositions, patient-specific profile etc.) as a carefully elaborated spectrum of measures for professional care that promotes the mental and physical health of an individual concept.

➣ Considering the sensitive ethical aspects of the laboratory medicine and biobanking, robust information governance of the database and high quality of ethical standards are the prerequisites for the successful implementation of PPPM in health care systems.

➣ Currently, bio-banking is facing major viability challenges. These critical problems may be optimally solved by relevant professional groups with complementary expertise such as the International Federations of Laboratory Medicine, EPMA and ESBB followed by adequate decisions of policymakers resulting in the creation of a robust juristic platform.

➣ Standardisation of health care services in Europe is a strategic issue for policymakers after detailed consultations with health care professionals.

➣ Professional education: we need to develop a new culture among experts in order to promote the multidisciplinary character of predictive, preventive and personalised medicine and concomitantly to advance currently deficient health care services. The innovative PPPM-related educational programmes for professionals should be prioritised in the *Common Strategic Framework* (also called as the New European Framework Programme Horizon 2020) as well as by other global and topic-relevant national programmes.
